# Sperm Membrane Stability: In-Depth Analysis from Structural Basis to Functional Regulation

**DOI:** 10.3390/vetsci12070658

**Published:** 2025-07-11

**Authors:** Shan-Hui Xue, Bing-Bing Xu, Xiao-Chun Yan, Jia-Xin Zhang, Rui Su

**Affiliations:** 1 College of Animal Science, Inner Mongolia Agricultural University, Hohhot 010018, China; nndxsh@163.com (S.-H.X.);; 2 Sino-Arabian Joint Laboratory of Sheep and Goat Germplasm Innovation, Hohhot 010018, China; 3 Inner Mongolia Key Laboratory of Sheep & Goat Genetics Breeding and Reproduction, Hohhot 010018, China

**Keywords:** membrane stability, sperm maturation, protein, reproduction

## Abstract

Sperm membrane stability is a key factor in determining sperm viability and fertilization capability, with broad implications ranging from basic reproductive biology to livestock breeding practices. This comprehensive review examines the structural and functional mechanisms underlying sperm membrane integrity, including defensive barrier functions, potentiometric ion channel regulation, and motility modulation that collectively optimize sperm survival, motility, and fertilization potential. Environmental factors such as temperature fluctuations, abnormal pH levels (outside the optimal 7.2–8.2 range), pathological conditions, and hormonal imbalances can compromise membrane stability by inducing oxidative stress and protein denaturation. Key regulatory proteins, notably NPC2 for cholesterol homeostasis, Flotillin proteins for lipid raft organization, and Annexin V for membrane repair mechanisms, demonstrate essential roles in maintaining structural integrity. In livestock reproduction, membrane stability research facilitates the optimization of cryoprotectant formulations and freezing protocols, resulting in 15–25% improvements in post-thaw sperm survival rates and enhanced artificial insemination success. These findings provide valuable insights for advancing assisted reproductive technologies and improving reproductive efficiency in animal husbandry.

## Simple Summary

Sperm health is crucial for animal reproduction and livestock breeding. The outer membrane of sperm cells acts like a protective shell that must remain stable for successful fertilization. This review examines what keeps sperm membranes healthy and what can damage them. Key findings show that three main systems help maintain membrane stability: cholesterol balance, lipid organization, and membrane repair. Environmental stress like temperature changes and wrong pH levels can damage these membranes. In livestock breeding, understanding membrane biology has led to better sperm freezing methods, improving success rates by 15–25%. This knowledge helps farmers and breeders improve animal reproduction efficiency.

## 1. Introduction

Livestock reproduction is central to global agricultural productivity, and sperm quality is a key determinant of success in artificial insemination and other assisted reproductive technologies. Among the various factors influencing sperm functionality, membrane stability has emerged as a critical parameter that directly impacts sperm viability, motility, and fertilization capacity [1]. Despite significant advances in reproductive biology, fundamental questions regarding the molecular mechanisms governing sperm membrane stability in livestock species remain incompletely understood.

Spermatozoa are specialized male reproductive cells responsible for transmitting genetic information. While their structure is generally conserved, species-specific variations exist across taxa. Spermatogenesis involves complex coordination between somatic and germ cells within seminiferous tubules, encompassing multiple stages of proliferation, meiosis, and differentiation [2,3]. During terminal sperm development, the nucleus undergoes extensive remodeling to form sperm-specific organelles and structures [4]. The fundamental sperm architecture comprises a head containing genetic material and an acrosome, connected via the centriole to a tail subdivided into middle, principal, and end pieces responsible for energy production and propulsion. The sperm membrane functions as an external barrier that not only protects internal sperm structures but also actively participates in regulating sperm maturation, capacitation, and fertilization processes. This membrane consists of a phospholipid bilayer embedded with diverse proteins and glycan chains that maintain membrane fluidity and stability [5]. Furthermore, environmental changes within the female reproductive tract critically affect sperm membrane fluidity at molecular and cellular levels, thereby facilitating sperm–oocyte interactions [6,7]. Pioneering ultrastructural studies by Åke Franzén and Björn Afzelius using electron microscopy provided foundational insights into sperm architecture and function [8]. However, the current understanding of sperm membrane stability mechanisms presents several critical knowledge gaps. First, the integrative effects of multiple environmental factors on membrane stability remain poorly characterized. Second, species-specific variations in key regulatory proteins and their functional mechanisms have not been systematically compared across livestock species. Third, the translation of fundamental research findings into practical improvements for reproductive technologies requires further development. These limitations are particularly problematic given the economic importance of optimizing reproductive efficiency in livestock production systems. Recent advances in molecular biology and multi-omics approaches have created unprecedented opportunities to address these knowledge gaps comprehensively. While existing reviews have focused primarily on human reproductive medicine or individual molecular mechanisms, a systematic synthesis integrating structural biology, environmental factors, and practical applications in livestock reproduction is urgently needed.

Therefore, this review aims to: (1) comprehensively analyze sperm membrane structural features and stability mechanisms; (2) systematically examine environmental and physiological factors affecting membrane stability; (3) elucidate the molecular functions of key regulatory proteins; and (4) explore the translational potential of research findings for improving livestock reproductive technologies. By providing this integrated perspective, we seek to advance both fundamental understanding and practical applications of sperm membrane stability research in animal reproduction.

## 2. Overview of Sperm Membrane Stability

**Figure 1:**
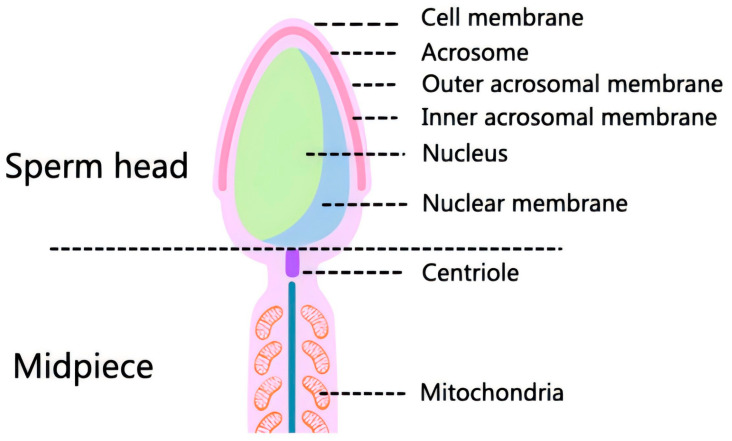
Structural model of sperm head and middle section.

The stability of sperm membranes is intrinsically linked to sperm structure and function. Membrane integrity directly affects sperm motility, capacitation ability, and fertilization potential, making it a fundamental determinant of reproductive success. The sperm membrane consists of a phospholipid bilayer with embedded proteins (Figure 1). Phospholipids comprise the primary lipid layer, conferring both structure and fluidity to the membrane. Meanwhile, embedded proteins facilitate substance transport, signal transmission, and sperm–egg interactions [1]. The sperm membrane also has an extracellular layer of glycolipids and glycoproteins, which is important for sperm–egg recognition. The head of the spermatozoon comprises several different membrane structures, including the outer plasma membrane, the outer and inner acrosomal membranes, and the nuclear membrane, which surrounds the nucleus. During fertilization, the plasma membrane of the spermatozoon fuses with the outer acrosomal membrane, which is a key step in the union of the spermatozoon with the egg [9]. However, the plasma membrane overlying the equatorial segment of the spermatozoon and the outer acrosomal membrane remain intact and do not undergo membrane fusion events during the initial stages of sperm–egg interaction. This phenomenon may reveal their unique functions during fertilization. Additionally, the intra-acrosomal membrane is secluded from other membranes throughout fertilization, suggesting that it may play a special role in this process [10]. The specificity of these membrane structures may be related to their role in sperm–egg recognition, binding, and subsequent fusion. Sperm membranes contain high levels of unsaturated fatty acids, which help to maintain membrane fluidity but also make it susceptible to damage by reactive oxygen species (ROS). To counteract this, antioxidants such as astaxanthin play a crucial role in protecting the sperm membrane from oxidative stress, thereby preserving sperm function and viability [11]. During fertilization, specific protein complexes in the sperm membrane, such as CatSper channelosomes, are critical for sperm activation and direction of movement [12]. These channelosomes contain channel as well as transporter proteins, further highlighting the complexity of the sperm membrane. Sperm membrane proteins, such as Izumo1, Spaca6, and Tmem81, form a specialized complex and act as a bridge during sperm–egg binding [13]. The interaction of these proteins increases the specificity of sperm–egg binding and efficiency of fertilization [14]. Thus, the sperm membrane not only protects the sperm and improves sperm quality, but also ensures precise and efficient transfer of genetic material to the next generation [15].

## 3. Structural Features of the Sperm Membrane

### 3.1. Sperm Membrane Potential and Ion Channels

After an animal ejaculates, the sperm are incapable of fertilization and must undergo capacitation [16]. This process usually occurs in the female reproductive tract, where spermatozoa need to recognize and respond to specific signals in the surrounding environment CatSper channel proteins [17]. Promoting sperm capacitation and fertilization capacity also requires fine regulation of Ca ion flux. During capacitation, the influx of intracellular bicarbonate ions (HCO^3−^) in sperm cells results from an increase in extracellular pH and an increase in bicarbonate concentration in seminal plasma and the female reproductive tract [18,19]. This change activates soluble adenylate cyclase, which promotes the production of cyclic adenosine monophosphate (cAMP) [20,21]. Increase in cAMP levels activates protein kinase A (PKA), triggering the phosphorylation of serine, threonine, and tyrosine residues on sperm proteins, which is a key step in sperm capacitation. Simultaneously, the precise modulation of sperm membrane potential and altered membrane composition promote the acrosome reaction [22]. In this series of changes, the regulation of Ca ion fluxes is crucial. The inward and outward flow of Ca ions occurs through specific channels and transport proteins in the sperm membrane, a process regulated by factors such as pH, cAMP levels, and PKA activity [23]. The dynamic balance of Ca ions not only affects sperm membrane stability but is also directly involved in the critical signaling pathway for sperm capacitation, ensuring that sperm can successfully enter the next physiological stage [24]. Sperm membranes have specific potentials that help sperm to quickly adapt to ionic changes in their environment and profoundly affect their viability and ultimately their ability to fertilize.

### 3.2. Sperm Membrane Fluidity

As sperm travel toward the egg, the cholesterol concentration in their membranes decreases, increasing membrane flexibility and fluidity—critical for initiating the acrosome reaction [25], which is essential for sperm to undergo the acrosome reaction. This reaction is a key step for sperm to penetrate the zona pellucida of the egg and fertilize it; the increase in membrane fluidity promotes the release of acrosomal enzymes and zona pellucida degradation, which ensures successful sperm penetration [9]. This process exemplifies the physical role of membrane fluidity and reveals its significance in the physiology of sperm fertilization. Higher membrane fluidity facilitates the dynamic distribution and function of key membrane proteins, such as ion channels and receptors [26]. These proteins are essential for sperm recognition and responsiveness to external stimuli. Increased membrane fluidity improves sperm sensitivity to these signals, accelerating the processes of sperm capacitation and activation. Regulation of sperm membrane fluidity is also critical for energy metabolism [27,28]. Increased membrane fluidity facilitates fatty acid β-oxidation through the mediation of transporter proteins such as SLC22A14 [29], thus providing a continuous and efficient supply of energy to the sperm. In addition to supporting sperm movement and fertilization, membrane fluidity also ensures sperm survival and competition in the female reproductive tract. Along with the physical properties and biological functions of spermatozoa, the regulation of sperm membrane fluidity also regulates sperm energy metabolism, motility, immune evasion, and molecular recognition and binding. During sperm maturation, changes in membrane fluidity are regulated by sperm maturation to ensure full fertilization. Therefore, a comprehensive study regarding the regulatory mechanisms of sperm membrane fluidity is critical to elucidate the process of sperm fertilization and improve the success rate of assisted reproduction techniques. However, increased membrane fluidity during cryopreservation can lead to premature capacitation (cryo-capacitation), which may compromise sperm viability in livestock applications. Understanding this balance is crucial for optimizing cryopreservation protocols.

### 3.3. Sperm Membrane Defense Barrier

**Figure 2:**
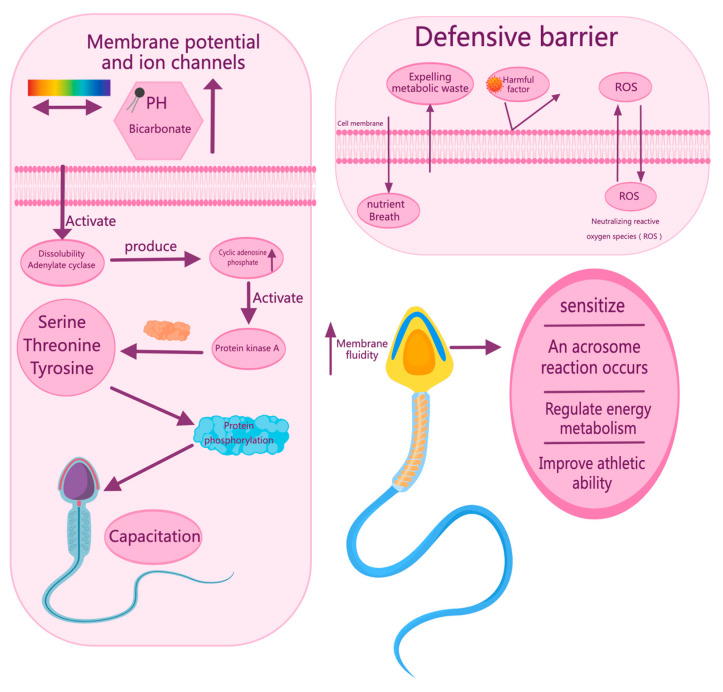
Biological characteristics of the sperm membrane.

The sperm membrane serves as a vital external barrier, shielding sperm cells from physical and chemical damage, preserving DNA integrity, and maintaining cellular morphology [30,31,32,33]. The selectivity and moderate permeability of the sperm membrane allows the entry of key nutrients and oxygen, while preventing the invasion of harmful factors, thus maintaining the stability and balance of the internal and external environment of sperm cells [34]. Specialized membrane components—such as the glycocalyx [35], complement regulators CD46 and CD55 [36], and FCRL3/MAIA [37]—help sperm evade immune recognition and reduce the risk of immune attack. These molecules form a protective layer on the surface of the spermatozoon and reduce nonspecific activation of the complement system, while acting as a bridge between the sperm and egg membranes to promote fertilization and membrane fusion. These specialized molecules provide comprehensive protection, supporting sperm survival, motility, and successful fertilization while ensuring accurate genetic transmission. The sperm membrane integrity plays a key role in preventing the accumulation of DNA damage. It effectively reduces DNA damage from oxidative stress by reducing ROS production and also promotes the effectiveness of DNA repair mechanisms, ensuring that sperm DNA damage can be repaired more efficiently by oocytes after fertilization [38]. Sperm activate multiple DNA repair mechanisms [39], such as nucleotide excision repair and base excision repair, during sperm formation; membrane integrity can support the optimum functioning of these repair mechanisms [40], thus reducing the risk of DNA damage. Therefore, sperm membrane integrity not only affects the survival and function of spermatozoa directly but also determines the occurrence of DNA damage and the ability to repair it (Figure 2).

## 4. Factors Influencing Sperm Membrane Stability

### 4.1. Physiological Factors (Temperature, pH)

Temperature fluctuations significantly affect sperm membrane stability. As temperatures rise, lipid phase transitions and protein denaturation occur within sperm membranes [41,42]. Consequently, enhanced thermal motion intensifies lipid molecular movement, increasing membrane fluidity [43]. In contrast, spermatozoa can suffer damage such as cold shock and osmotic pressure changes, thus compromising sperm membrane integrity. Marked temperature changes also triggers the sperm to produce large amounts of ROS, which attack polyunsaturated fatty acids on the sperm membrane, forming free radicals and producing malondialdehyde [44]. The accumulation of these substances ultimately damages the structure and function of the sperm membrane [45,46] resulting in a decrease in membrane stability. Various antioxidant mechanisms exist in organisms, including enzymatic and non-enzymatic antioxidants, which can neutralize excess ROS and protect spermatozoa from damage due to oxidative stress. Therefore, an in-depth understanding of the effects of temperature changes on sperm membrane stability, as well as the exploration of effective antioxidant strategies, are important for protecting sperm from damage resulting from environmental changes.

Changes in the pH of semen also affect membrane stability. The pH of semen usually ranges from 7.2 to 8.2, which has a positive effect on sperm motility [47]. However, semen pH varies among livestock species. Generally, the semen of cattle and sheep is weakly acidic to neutral, while that of horses tends to be more alkaline. These species-specific differences must be considered when developing optimal reproductive protocols. Abnormal pH levels may adversely affect the physiological function of sperm. pH changes indirectly affect sperm capacitation and motility. Different pH values of sperm capacitating fluid may affect the changes in Ca ion levels in the sperm cytoplasm, thus affecting various physiological activities of spermatozoa [48]. At low pH, the Na^+^/K^+^-ATPase activity of spermatozoa decreases, leading to reduced sperm motility and energy acquisition, which may be a potential mechanism of sterility in males [49]. Maintaining optimum pH is equally important during sperm cryopreservation [50]. Appropriate pH helps to maintain the stability of sperm cell membranes, thereby protecting sperm integrity and function [51]. pH balance also plays an important role in sperm metabolic processes, including regulation of sperm membrane potential and activation of adenylate cyclase synthesis, which promotes the elevation of cAMP levels and influences sperm energetic processes [52]. Thus, changes in semen pH affect the physiological function and the energized state of spermatozoa by influencing the stability of sperm membrane and metabolic processes.

### 4.2. Diseases and Hormone Levels

Infections or inflammatory conditions of the reproductive tract, such as epididymitis [53], seminal vesiculitis [54], and prostatitis [55], can damage sperm membranes and affect sperm viability [56]. These pathological conditions alter the sperm membrane by producing inflammatory mediators that interfere with the normal function of the spermatozoa. Changes in the levels of hormones, particularly sex hormones and thyroid hormones, significantly affect sperm membrane stability. Abnormally high prolactin levels inhibit the function of the hypothalamic–pituitary–gonadal (HPG) axis, reducing the release of gonadotropin-releasing hormone, which in turn decreases the secretion of luteinizing and follicle-stimulating hormone, thus affecting sex hormone synthesis and sperm production [57]. Abnormalities in thyroid function may affect the size of the testes and the proliferation and differentiation of interstitial and supportive cells by affecting the function of the HPG axis and hypothalamic–pituitary–thyroid axis. This may result in altered lipid and protein composition of sperm membranes, which can affect membrane stability and sperm function [58]. Therefore, maintaining sperm membrane stability requires a combination of avoiding reproductive tract infections, controlling the balance of hormone levels, and supporting the precise molecular mechanisms operating within the sperm cell. These factors work in coordination to safeguard sperm health and fertility. Understanding these environmental challenges to membrane stability leads us to examine the specific molecular mechanisms that cells have evolved to maintain membrane integrity under stress.

## 5. Key Proteins Maintaining Sperm Membrane Stability and Their Molecular Mechanisms

The stability of the sperm plasma membrane, which is crucial for ideal sperm movement and successful fertilization during the complex reproductive process, depends on the expression and function of specific proteins and genes. Any alterations in sperm plasma membrane can adversely affect sperm function. Therefore, a comprehensive study of the proteins and genes associated with the plasma membrane is essential for enhancing our understanding of sperm function and for developing methods to improve sperm quality and fertilization rates.

### 5.1. Cholesterol Homeostasis and the Role of NPC2 Protein

Maintaining cholesterol balance is crucial for sperm membrane stability. The Niemann–Pick C2 (NPC2) protein, a highly conserved molecule across species, plays a central role in cholesterol transport and regulation [59]. NPC2 protein can bind specifically to cholesterol molecules [60]; this property is essential for intracellular cholesterol transport and regulation. In sperm membranes, NPC2 protein plays a complex regulatory role in membrane stability. While NPC2 facilitates cholesterol efflux and redistribution within membrane domains, which can influence membrane fluidity and organization [61], its primary function appears to be maintaining cholesterol homeostasis. Studies have shown that NPC2 protein deletion significantly increases cholesterol loss from mouse sperm membranes, thus highlighting the critical role of NPC2 in maintaining cholesterol homeostasis in sperm membranes [62]. The regulation of cholesterol content by NPC2 is essential for proper sperm membrane function, as both excessive cholesterol accumulation and depletion can impair sperm capacitation and fertilization ability. While the precise mechanisms by which NPC2 regulates sperm membrane stability remain unclear, abnormal expression or function of this protein can disrupt membrane structure and impair fertilization capacity. Further studies are needed to reveal the precise role of NPC2 in animal reproductive health and its potential as a target for improving sperm quality.

### 5.2. Regulation of Lipid Rafts and Flotillin Proteins

The lipid raft proteins, Flotillin-1 and Flotillin-2, play crucial roles in maintaining sperm membrane stability and fluidity by forming specialized membrane microregion structures, known as lipid rafts [63]. Lipid rafts serve as platforms for signaling molecules and influence sperm maturation, motility, and fertilization, largely through the membrane-interacting domains of Flotillin proteins [64]. The distribution of Flotillins in the sperm membrane shows dynamic changes, which are closely related to the functional status of spermatozoa [65]. The membrane-binding process of Flotillins is finely regulated by post-translational modifications such as S-palmitoylation and N-myristoylation, which are essential for the precise localization and function of Flotillins in sperm membranes [66]. Although Flotillins have been studied in sperm membranes, their precise molecular roles and regulatory mechanisms require further investigation.

### 5.3. The Role of Annexin V in Sperm Membrane Repair

Annexin V is a calcium-dependent membrane-associated protein essential for maintaining membrane integrity. It participates in the Ca^2+^-triggered repair response to localized membrane damage caused by mechanical stress and extracellular Ca^2+^ influx. Annexin V can rapidly self-assemble into an ordered two-dimensional lattice at the site of injury in the presence of Ca^2+^ in response to the Ca^2+^ surge and promote membrane repair [67]. As a member of the membrane-bound protein family, Annexin V plays a key role in the integrity and fluidity of sperm membranes by specifically binding to phosphatidylserine and participating in the membrane repair process, thus stabilizing the membrane structure and preventing the leakage of internal contents. In addition, Annexin V may also regulate sperm motility and fertilization ability by influencing membrane dynamics and participating in signaling during sperm capacitation, thus affecting sperm–egg interactions [68]. In assisted reproductive technology, Annexin V improves the efficiency of gender-controlled semen production by immunizing magnetic beads to remove dead spermatozoa [69]. In the future, Annexin V can potentially serve as a promising biomarker for assessing sperm quality and fertility, providing new insights into reproductive biology and medicine.

### 5.4. Membrane Backbone Proteins and Sperm Structural Stability

The viability and function of spermatozoa are not only dependent on complex molecular mechanisms within their cells but are also closely related to the stability of their membrane skeleton proteins, which work together to maintain the structural integrity and function of spermatozoa [70]. Membrane backbone proteins form the supporting structures beneath the sperm cell membrane and have an important impact on the sperm membrane in terms of maintaining a specific morphology, regulating membrane fluidity, participating in motility, protecting the internal structure, participating in the capacitation and acrosome reactions, influencing fertilization, and playing a role in the sperm maturation process [71]. These proteins maintain membrane organization by arranging membrane components precisely and forming structural oligomers [72]. These proteins ensure that the precise morphology and mobility of sperm are maintained through interactions with sperm membrane lipids and proteins, thereby supporting sperm motility and key fertilization events, such as interactions with the egg zona pellucida and acrosome reaction. Furthermore, membrane skeleton proteins protect the internal organelles and DNA of the spermatozoa from external damage, particularly from challenges that may be encountered as the sperm passes through the female reproductive tract. They may also be involved in regulating sperm membrane remodeling and maturation, which is essential for sperm to achieve full fertilization. Thus, any abnormality or dysfunction of the membrane skeleton proteins may result in abnormal sperm morphology, reduced motility, or reduced fertilization capacity, thereby affecting the fertilization rate of sperm. Understanding how these proteins contribute to spermatogenesis, sperm maturation, and function is vital for advancing our knowledge of sperm biology.

### 5.5. Protective Effect of Heat Shock Proteins on Sperm Membranes

Heat shock proteins (HSPs) are produced by cells in response to stress conditions such as high temperature, oxidative stress, or chemical stimuli. They primarily aid cells in protecting and repairing other proteins, thus preventing protein misfolding and aggregation, to maintain intracellular environment stability. In sperm cells, heat shock proteins (HSPs) are essential for maintaining membrane stability and function. For example, HSP70 [73] and HSP90 [74] are involved in the correct folding and repair of proteins in sperm cells, which is essential for the function of sperm membrane proteins, sperm morphology, motility, and fertilization ability. Under stress conditions, HSPs protect sperm membranes from damage, such as lipid peroxidation and oxidative-stress induced protein denaturation. Furthermore, HSPs help maintain sperm membrane fluidity, which is important for sperm capacitation, the acrosome reaction, and fusion with the egg. HSPs may also regulate the expression and distribution of sperm membrane proteins during sperm maturation, thus affecting sperm maturation and fertilization ability. By stabilizing membranes and associated proteins, HSPs preserve sperm viability and motility—key to successful fertilization. In assisted reproductive technology, the protective effect of HSPs may help improve sperm viability and fertilization success after recovery.

### 5.6. Phosphatases, Kinases, and the Regulation of Sperm Membrane Dynamics

Phosphatases and kinases are crucial enzymes that regulate proteins involved in cytoskeletal integrity and membrane stability by regulating the phosphorylation state of proteins. These enzymes play a crucial role in regulating sperm membrane stability. For example, during sperm capacitation, an increase in HCO^3-^ and Ca^2+^ levels can activate G proteins and adenylyl cyclase, which in turn generate cAMP as a second messenger. cAMP increases activate PKA, which further phosphorylates target proteins and alters their activity, thereby affecting key processes, such as sperm hyperactivation motility, acrosome reaction, and sperm–egg binding [75]. In addition, lysophosphatidic acid acylcholinesterase (lysophospholipase) also plays a significant role in sperm membrane stability [76]. It maintains membrane phospholipid homeostasis by removing potentially membrane-damaging lysophospholipids, protecting and stabilizing the membrane structure, and is essential for sperm membrane integrity and function. Sperm membrane stability is also influenced by various other factors, including glycosylation modifications of proteins and GPI-anchored modifications. For instance, the family of O-glycosylation-modified mucins is differentially expressed during spermatogenesis, and their absence leads to impaired spermatogenesis and infertility [77]. GPI-anchored glycoproteins, such as hyaluronidase (SPAM1/PH-20) and plasma membrane calcium-ATPase 4, also play important roles. By binding to the cell membrane, they influence processes such as sperm survival and capacitation in the female reproductive tract. Sperm membrane stability is maintained through a complex interplay of enzymes and protein modifications. These factors coordinate to ensure that spermatozoa are mature and functionally capable of adapting to the complex reproductive environment, ultimately leading to fertilization. To improve sperm quality and function, future studies could further explore the mechanisms of action of these enzymes and protein modifications and how they function in concert to maintain sperm membrane stability and promote sperm capacitation. The key regulatory proteins and their mechanisms are summarized in Table 1, which provides an integrated overview of the molecular machinery governing sperm membrane stability.

## 6. The Critical Role of Sperm Membrane Stability in Livestock Reproduction

Membrane stability is an important indicator of sperm cell function and integrity in livestock reproduction technology; it has a significant impact on reproductive performance, growth and development, and the overall health of livestock. A comprehensive understanding of membrane stability has led to the development of improved cryoprotectants and freezing protocols to support sperm viability during storage and thawing [78]. Lipid composition of sperm membranes plays an important role in their stability during freezing and thawing [79]. Ram sperm membranes are particularly sensitive to cryopreservation due to their low sterol content. Enhancing this content—especially through the incorporation of cholesterol and desmosterol—can significantly improve membrane stability [80]. Maintaining sperm membrane stability in the context of animal husbandry reproductive technology is critical to improving the success of assisted reproductive techniques, such as artificial insemination [81,82]. Applications are focused on enhancing sperm membrane stability, further optimizing cryopreservation protocols, increasing post-thaw survival and improving fertility outcomes. In addition, the integrated application of advanced technologies such as multi-omics analysis (including transcriptomics and proteomics) has provided insights into the molecular mechanisms of sperm membranes subjected to freezing damage [83]. These techniques mainly focus on the molecular changes in sperm membrane stability and abnormal regulation of membrane protein function under freezing stress. Transcriptomic analysis has been successfully identified genes differentially expressed during freezing, which may be directly related to the maintenance of sperm membrane structure and function [84]. At the proteomics level, studies have revealed changes in the expression levels or post-translational modification status of specific proteins [85], These proteins often serve as sensitive markers of sperm membrane cryoinjury, which have evolved our understanding regarding the mechanism of membrane damage during sperm cryopreservation, providing potential targets for optimizing cryopreservation techniques and developing conservation strategies. This approach not only supports the reproduction of genetically superior animals but also contributes to the conservation of endangered species through effective breeding programs, playing a crucial role in the future of animal reproductive technology. Species-specific differences in membrane lipid composition significantly affect cryoresistance. For example, ram sperm membranes contain lower sterol levels compared to bull sperm, making them more susceptible to cryoinjury. Understanding these species variations is essential for developing tailored cryopreservation strategies for different livestock species.

## 7. Conclusions

This review provides a detailed synthesis of the diverse mechanisms underlying sperm membrane stability and their critical role in improving reproductive success in livestock. Key findings demonstrate that membrane stability is fundamentally regulated by three primary mechanisms: cholesterol homeostasis via NPC2 protein, lipid raft organization through Flotillin proteins, and membrane repair mediated by Annexin V. Environmental factors, particularly temperature fluctuations and pH deviations from the optimal 7.2–8.2 range, significantly compromise membrane integrity through oxidative stress induction. The integration of these findings into livestock reproduction has yielded substantial improvements, with optimized cryoprotectant formulations and freezing protocols achieving 15–25% enhancement in post-thaw sperm survival rates. Nonetheless, significant knowledge gaps remain, particularly regarding species-specific differences in protein function and the combined impact of environmental stressors. Future research should prioritize: (1) developing species-specific cryoprotectant formulations based on membrane lipid profiles; (2) establishing real-time membrane stability monitoring systems using fluorescent biomarkers; (3) creating predictive algorithms that integrate temperature, pH, and oxidative stress parameters for reproductive protocol optimization; and (4) investigating the role of membrane microdomains in species-specific fertilization mechanisms through advanced microscopy techniques.

## Figures and Tables

**Table 1:** Key proteins regulating sperm membrane stability and their functional mechanisms.

Protein/System	Primary Function	Molecular Mechanism	Key Role in Fertility
NPC2	Cholesterol homeostasis	Cholesterol binding and transport; membrane domain redistribution	Essential for capacitation and membrane fluidity regulation
Flotillin-1/2	Lipid raft organization	Formation of membrane microdomains; protein scaffolding	Supports signaling pathways and sperm maturation
Annexin V	Membrane repair	Ca^2+^-dependent membrane resealing; phosphatidylserine binding	Maintains structural integrity under stress
HSP70/90	Protein protection	Molecular chaperone activity; prevents protein misfolding	Preserves membrane protein function
Membrane skeleton proteins	Structural stability	Protein oligomerization; membrane-cytoskeleton interactions	Maintains cell morphology and motility
Phosphatases/Kinases	Dynamic regulation	Protein phosphorylation/dephosphorylation cycles	Controls capacitation and signaling cascades

## Data Availability

No new data created in this manuscript.

## Abbreviations

The following abbreviations are used in this manuscript:

ROS	reactive oxygen species
cAMP	cyclic adenosine monophosphate
HPG	hypothalamic–pituitary–gonadal
NPC2	Niemann–Pick C2
HSPs	Heat shock proteins
